# Expression of functional toll like receptor 4 in estrogen receptor/progesterone receptor-negative breast cancer

**DOI:** 10.1186/s13058-015-0640-x

**Published:** 2015-09-22

**Authors:** Meliha Mehmeti, Roni Allaoui, Caroline Bergenfelz, Lao H. Saal, Stephen P. Ethier, Martin E. Johansson, Karin Jirström, Karin Leandersson

**Affiliations:** Center for Molecular Pathology, Department of Translational Medicine, Lund University, SUS Jan Waldenströmsgata 59, 20502 Malmö, Sweden; Division of Oncology and Pathology, Department of Clinical Sciences, Lund University, Lund, Sweden; Department of Pathology and Laboratory Medicine, Hollings Cancer Center, Medical University of South Carolina, Charleston, SC USA

## Abstract

**Introduction:**

Toll-like receptors (TLRs) are a family of pattern recognition receptors that are expressed on cells of the innate immune system. The ligands can be pathogen derived (pathogen associated molecular patterns; PAMPs) or endogenous (damage associated molecular patters; DAMPs) that when bound induces activation of nuclear factor kappa B (NF-κB) and transcription of pro-inflammatory genes. TLRs have also been discovered in various malignant cell types, but with unknown function.

**Methods:**

In this study we performed a detailed analysis of TLR and co-receptor expression pattern and function in breast cancer. Expression patterns were examined using real-time quantitative polymerase chain reaction (RT-qPCR) and immunohistochemistry (IHC) on three estrogen receptor-positive (ER^+^) and four estrogen receptor/progesterone receptor-negative (ER^−^/PR^−^; ER/PR-negative) breast cancer cell lines, and a breast cancer cohort consisting of 144 primary breast cancer samples. The function was investigated using *in vitro* assays comprising PAMP/DAMP-stimulation, downstream signaling and TLR-silencing experiments.

**Results:**

We found that TLR4 was expressed in a biologically active form and responded to both PAMPs and DAMPs primarily in ER/PR-negative breast cancers. Stimulation of TLR2/4 *in vitro* induced expression of pro-inflammatory genes and a gene expression analysis of primary breast cancers showed a strong correlation between TLR4 expression and expression of pro-inflammatory mediators. In line with this, TLR4 protein expression correlated with a decreased survival.

**Conclusions:**

These findings suggest that TLR4 is expressed in a functional form in ER/PR-negative breast cancers. Studies regarding TLR4-antagonist therapies should be focusing on ER/PR-negative breast cancer particularly.

**Electronic supplementary material:**

The online version of this article (doi:10.1186/s13058-015-0640-x) contains supplementary material, which is available to authorized users.

## Introduction

Breast cancer is the most common form of cancer among women today [1]. The prognosis of breast cancer patients varies depending on the breast cancer subtype. Clinical breast cancer classification is based on expression of various immunohistochemical markers, with the hormone receptors being the most important. One of the worst prognosis subtypes is the triple-negative (TN) breast cancer subtype, where the malignant cells lack expression of the hormone receptors, estrogen receptor (ER) and progesterone receptor (PR), and human epidermal growth factor receptor 2 (Her2) (ER^−^PR^−^Her2^−^). The treatment options are few for patients with TN breast cancer [2–4].

Toll-like receptors (TLRs) are a family of receptors that are expressed on innate immune cells [5]. They are part of the pattern recognition receptor (PRR) family and recognize molecular patterns from pathogens (pathogen-associated molecular patterns; PAMPs) or from endogenous stress-induced proteins (damage-associated molecular patterns; DAMPs) [6–9]. Signaling via TLRs leads to activation of nuclear factor kappa B (NFκB) and a subsequent expression of pro-inflammatory genes [10]. There are 10 different TLRs (TLR1-10) in humans, and these are divided into two subgroups depending on cellular localization; on the surface of the cell (TLR1, TLR2, TLR4, TLR5 and TLR6), or in vesicles such as endoplasmic reticulum, endosomes or lysosomes (TLR3, TLR7, TLR8 and TLR9). Lately, expression of different TLRs has been described in various malignancies, although their function is as yet unclear [5, 11, 12].

TLR2 and TLR4 respond to the typical PAMP from Gram-negative bacteria, lipopolysaccharide (LPS). Different variants of LPS (from *Escherichia coli* and *Salmonella typhimurium*) induce different TLR-intracellular signals [13]. DAMPs can also bind to and activate TLR2 or TLR4, and two endogenous ligands that are well-described are HMGB1 and S100A9 [14–19]. To signal via TLR2 or TLR4, different ligands may also require the co-receptors CD14 or MD2 [20–23]. All TLR ligands initiate activation of NFκB, but also mitogen-activated protein kinase (MAPK) pathways that affect protein translation and processing rather than transcription can be activated [24]. TLR4 has previously been shown to be expressed in breast cancer [25, 26].

The transcriptional factors ERα and NFκB are synergistically interrelated, although their exact interactions are unknown [10, 27–31]. NFκB is a transcriptional factor that induces a wide array of pro-inflammatory mediators and is also related to several oncogenic processes [32]. Both ER and NFκB have previously been shown to attenuate each other in different ways. In line with this observation, ER^−^ breast cancers have a stronger pro-inflammatory phenotype and microenvironment. NFκB has even been shown to downregulate ERα expression in breast cancer cells [29], but there is no direct proof that constitutive NFκB would generate ER^−^ breast cancers in general. On the other hand, a recent positive synergy between ER and NFκB was published, where TNFα and estrogen were shown to remodulate the ERα-promoter landscape in an NFκB and FoxA1 dependent manner resulting in an altered gene expression pattern [33].

In this study we performed an analysis of TLR expression patterns and function in breast cancer. Using a carefully validated TLR4-specific antibody for immunohistochemistry (IHC), we found that TLR4 protein expression was primarily present in breast cancers of ER/PR-negative phenotype. Using three cell lines of ER^+^ phenotype and four cell lines of the TN phenotype, we further showed that the expressed TLR4 was biologically active and hence responding to both PAMPs and DAMPs, primarily in the TN breast cancer cell lines. Finally, TLR4 protein expression correlated with a decreased survival in a cohort of 144 primary breast cancer patients. We propose that novel therapies targeting TLR4 may be of value, in particular in ER/PR-negative breast cancers.

## Methods

### Cell culture

The human breast cancer cell lines MCF-7, T47D, MDA-MB-231 and MDA-MB-468 were purchased from ATCC and were cultured in RPMI 1640 medium supplemented with 10 % fetal bovine serum (FBS) (Biosera, Boussens, France), 1 % sodium pyruvate, 1 % HEPES and penicillin/streptomycin (100 U/ml and 100 μg/ml respectively); CAMA-1 (also purchased from ATCC) was cultured in MEM/EBSS supplemented with 10 % FBS and penicillin/streptomycin, and SUM-149 and SUM-159 were cultured in F-12 HAM’S medium supplemented with 5 % FBS, 1 mM L-Glutamine, 1 μg/ml hydrocortisone (BD BioScience, San Diego, CA, USA) and 5 μg/ml insulin (Novo Nordisk A/S, Måløv, Denmark). The SUM-149 and SUM-159 cell lines were produced by Professor S Ethier. Media and supplements were purchased from Thermo Scientific HyClone (South Logan, UT, USA) unless otherwise stated.

### Compounds and cytokine analysis

LPS was purchased from Sigma Aldrich (St Louis, MO, USA) and originated from *S. Typhimurium* (LPS1) and *E. Coli* (LPS2)*,* respectively. All stimulations were performed for a total of 6 h except for rhS100A9 (20 h). IL-1β and HMGB1 was from R&D Systems. Recombinant human S100A9 (rhS100A9) was a gift from Active Biotech AB and a detailed description on endotoxin-free S100A9 generation and purification has been published previously [15] and was used in the presence of calcium and zinc (Ca^2+^ ≥200 μM; 10 μM ZnCl_2_ [34, 35]). Supernatants from stimulated or siRNA transfected cells were harvested and analyzed using human inflammatory cytokine cytometric bead array (CBA; BD Biosciences, San Diego, CA, USA) according to the manufacturer’s instructions or using IL-6 and IL-8 Quantikine ELISA (R&D Systems, Minneapolis, MN, USA). Annexin V-allophycocyanin (APC) and propium iodide (PI) staining was performed according to the manufacturer’s instructions (BD Biosciences). The cycloheximide (CHX) experiments (Sigma Aldrich) where performed by adding 10 μg/ml CHX, with or without 100 ng/ml LPS for 6 h.

### Preparation of necrotic cell supernatant (NCS)

Confluent monolayers of MDA-MB-231 cells were harvested by trypsinization and 3.2 × 10^6^ cells were resuspended in 2 ml serum-free RPMI-1640 medium. Necrosis was induced by performing three freeze-thaw cycles and NCS was separated from the necrotic cell pellet by centrifugation.

### Tissue microarray (TMA) and immunohistochemistry

The breast cancer cohort analyzed in this study consists of 144 patients diagnosed with invasive breast cancer at Skåne University Hospital, Malmö, Sweden, between 2001 and 2002. The cohort and TMA have previously been described in detail [36–38] and [39]. TMA sections of 4 μm thickness were mounted onto glass slides and deparaffinized followed by antigen retrieval using the PT-link system (DAKO, Glostrup, Denmark) and stained in an Autostainer Plus (DAKO) with the EnVisionFlex High pH-kit (DAKO). Antibody used for TLR4 IHC was anti-TLR4 NB100-56566 at 1:250 (Novus Biologicals, Littleton, CO, USA). TLR4 expression in TMA tumor samples was estimated as cytoplasmic staining intensity (0 = negative, 1 = weak, 2 = moderate, 3 = strong intensity and 4 = very strong intensity).

### Ethical considerations

Ethical permit was obtained from the regional ethical committee at Lund University (Dnr 447/07), waiving the requirement for signed informed consent. Patients were offered to opt out of research. Ethical permission for using blood from healthy blood donors was obtained from the regional ethical committee at Lund University (Dnr 2012/689).

### Gene expression profile array

The publicly available database R2: microarray analysis and visualization platform [40]; Tumor breast EXPO-351 was used for gene expression profile analysis.

### Quantitative real-time PCR (RT-qPCR)

RNeasy Plus kit was used to extract total RNA according to the manufacturer’s instructions (Qiagen, Hilden, MD, USA). Random hexamers and the M-MuLV reverse transcriptase enzyme (Thermo Scientific) was used and quantitative real-time PCR (RT-qPCR) were performed in triplicates for the genes analyzed using Maxima SYBR Green/Rox (Thermo Scientific) according to the manufacturer’s instructions. RT-qPCR analysis was performed on the Mx3005P QPCR system (Agilent Technologies, Santa Clara, CA, USA) and the relative mRNA expression was normalized to *YWHAZ*, *UBC* and *SDHA* and calculated using the comparative cycle threshold (Ct) method [41]. For primers see Additional file 1: Table S1.

### Transient transfections

siRNA transfections were performed using Lipofectamine 2000 (Invitrogen, Carlsbad, CA, USA): 2 μM of the following silencer select siRNA oligonucleotides from Ambion (Carlsbad, CA, USA) were used; Silencer Select Negative Control #2: 4390846, siTLR2 #1: s168, siTLR2 #2: s170, siTLR4 #1: s14194, siTLR4 #2: s14195. Analyses were performed 48 h and 72 h post transfection. For luciferase assays, breast cancer cells were co-transfected using Lipofectamine 2000 with a total of 0.6 μg pNFκB-luciferase (BD Biosciences) and 0.06 μg TK-renilla-luciferase (Promega, Madison, WI, USA) plasmids and was subsequently analyzed using Dual-Luciferase Reporter System (Promega). For TLR4 transfections breast cancer cells were transfected using Lipofectamine 2000 with a total of 1.0 μg pDUO-MD2/hTLR4 or pUNOI-hTLR4-GFP (Invivogen, San Diego, CA, USA) per 24 wells for 72 h or 48 h, respectively, and was subsequently analyzed using immunofluorescence (×40 magnification) or ELISA as described in the figure legends.

### Statistical analyses

Graph Pad Prism software was used to perform analysis of variance (ANOVA) or Students *t* test for the *in vitro* experiments as indicated. Spearman's Rho and the chi-square (*χ*^2^) test was used for correlation analysis and Kaplan-Meier analysis with the log-rank test was used to illustrate differences in survival. All statistical tests were two sided and *P* ≤0.05 was considered significant. Calculations were performed with IBM SPSS Statistics version 19.0 (SPSS Inc).

## Results

### TLR and co-receptor mRNA expression pattern in breast cancer cell lines

**Fig. 1:**
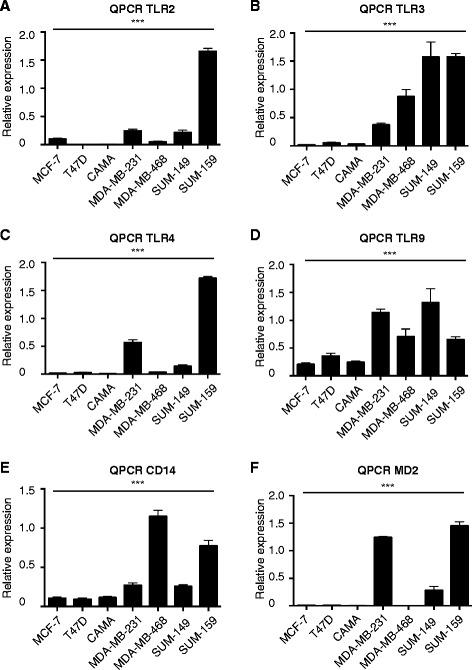
Breast cancer cell line mRNA expression levels of Toll-like receptor 2 (*TLR2*)*, TLR3, TLR4, TLR9* and co-receptors *CD14* and *MD2*. **a**-**f** The relative expression of indicated mRNA using quantitative real-time PCR (*QPCR*) on mRNA from the cell lines indicated. *Error bars* standard error of the mean: n = 6 − 9; ***P <0.001 (analysis of variance)

Most studies of TLRs in breast cancer have been performed using the ER^+^ cell line MCF-7 and the TN cell line MDA-MB-231 [5]. To our knowledge, a detailed comparison between ER^+^ and TN cell lines or cancers has not been published. We initially performed a broad analysis on TLR and TLR2/4 co-receptor (CD14 and MD2) mRNA expression patterns in various breast cancer cell lines. We used three cell lines with an ER^+^PR^+^ phenotype (MCF-7, T47D and CAMA-1) and four with an ER^−^PR^−^Her2^−^ (TN) phenotype (MDA-MB-231; MDA-MB-468, SUM-149 and SUM-159). As shown in Fig. 1a-c, *TLR2*, *TLR3* and *TLR4* were preferentially expressed in the TN cell lines while *TLR9* was more generally expressed (Fig. 1d). Only MDA-MB-468 had low/absent mRNA expression levels of *TLR2* and *TLR4* of the TN cell lines. Similarly, the TLR4 co-receptors *CD14* and *MD2* were expressed primarily in the TN cells lines (Fig. 1e, f). Again, the TN cell line MDA-MB-468 stood out with high *CD14* mRNA expression levels, but low *MD2* levels (Fig. 1e, f). This means that three out of the four TN breast cancer cell lines had the necessary proteins for a functional TLR4 signal to occur.

### The TLRs are functional and activation promotes expression of pro-inflammatory genes

**Fig. 2:**
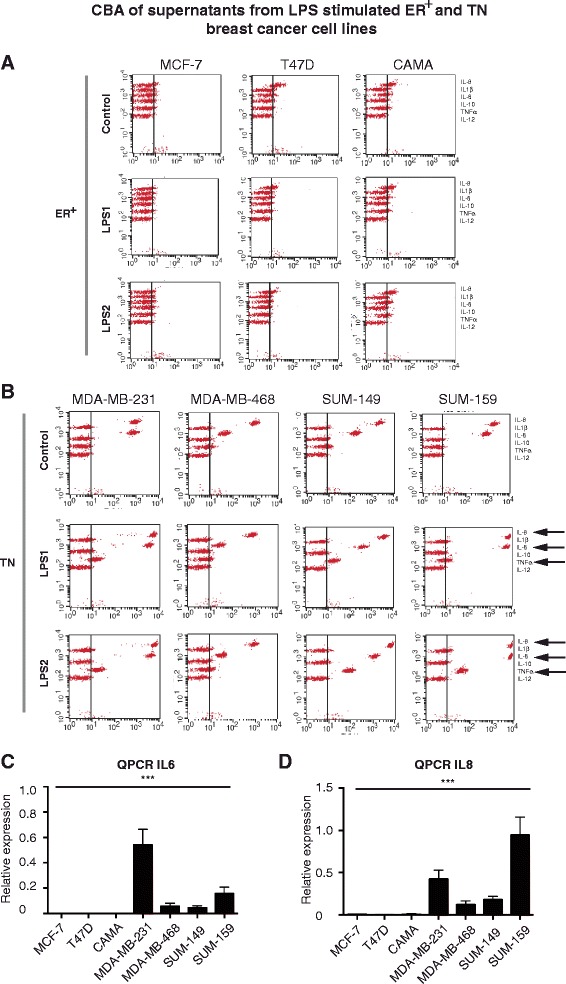
Lipolysaccharide (*LPS*) induced cytokine release in human breast cancer cells in vitro. Release of cytokines by breast cancer cells of estrogen receptor-positive (*ER* ^*+*^) origin (**a**) and triple-negative (*TN*) origin (**b**) was analyzed using cytokine bead array (*CBA*). Unstimulated breast cancer cells of TN origin (**b**) produce IL-6 and IL-8 at high levels. LPS1 (from *S. Typhimurium*) and LPS2 (from *E. Coli*) stimulation for 6 h induced release of IL-8, IL-6 and TNFα from indicated breast cancer cell lines. At least three experiments were performed for each cell line. The relative mRNA expression of *IL-6* (**c**) and *IL-8* (**d**) in unstimulated cells was measured using quantitative real-time PCR (*QPCR*). At least five experiments were performed for each cell line. *Error bars* standard error of the mean; ***P <0.001 (analysis of variance)

**Fig. 3:**
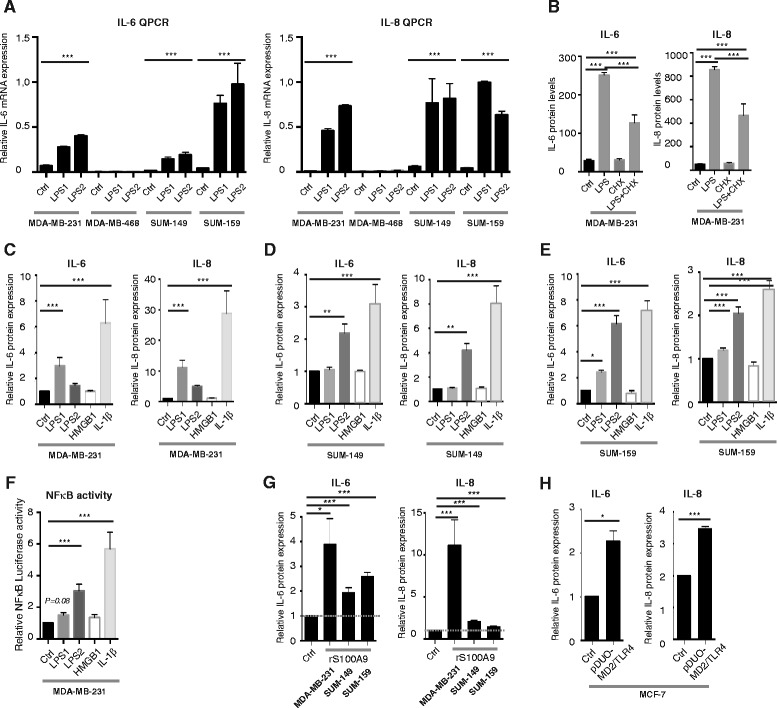
Pathogen-associated molecular patterns (PAMPs) and damage-associated molecular pattern (DAMPs) induce cytokine expression in human breast cancer cells in vitro. **a** Relative expression of *IL-6* and *IL-8* mRNAs after 6 h stimulation with lipopolysaccharide 1 (*LPS1*) and LPS2, using quantitative real-time PCR (*QPCR*) on mRNA from the cell lines indicated; n = 6. *Error bars* standard error of the mean (SEM); ***P <0.001 (analysis of variance (ANOVA)). **b** Release of IL-6 and IL-8 after stimulation with LPS1 and cycloheximide for 6 h, using ELISA on supernatants from stimulated MDA-MB-231 cells; n = 3. **c-e** Relative release of IL-6 and IL-8 after stimulation with LPS1, LPS2, the DAMP HMGB1 or IL-1β for 6 h, using ELISA on supernatants from stimulated MDA-MB-231 cells (**c**), SUM-149 cells (**d**) and SUM-159 cells (**e**); n = 10. *Error bars* SEM; *P <0.05, ***P <0.001 (ANOVA). **f** Dual luciferase reporter assays of MDA-MB-231 cells transfected with an NFκB reporter. TK-Renilla was co-transfected as control (*Ctrl*). LPS1, LPS2, HMGB1 or IL-1β was added to stimulate NFκB activity as described in “Methods”; n = 14. *Error bars* SEM; ***P <0.001 (ANOVA). **g** The relative release of IL-6 and IL-8 after stimulation with the DAMP S100A9 for 20 h, using ELISA on supernatants from stimulated MDA-MB-231, SUM-149 and SUM-159 cells; n = 6. *Error bars* SEM. *P <0.05, ***P <0.001 (ANOVA). **h** Transient transfection of pDUO-MD2/hTLR4 for 72 h in MCF-7 cells induces release of both IL-6 and IL-8 measured by ELISA; n = 6. *Error bars* SEM; *P <0.05, ***P <0.001 (Students *t* test)

To investigate whether the expressed TLRs were functional in the breast cancer cells following LPS stimulation, we analyzed the expression levels of some pro-inflammatory genes that are known targets for NFκB. The pro-inflammatory cytokines IL-6 and IL-8 were expressed at both protein (Fig. 2a and b) and mRNA (Fig. 2c and d) levels and only in the TN breast cancer cells but not the ER^+^ breast cancer cells. The TLR2/4-ligand LPS induces different TLR downstream signaling pathways when originating from different bacterial strains [13]. When the breast cancer cells were stimulated with LPS for 6 h (LPS1 from *S. Typhimurium* and LPS2 from *E. Coli*), we could see that IL-6, IL-8 and TNFα were induced by both LPS1 and LPS2 in the MDA-MB-231, SUM-149 and SUM-159 cell lines, but not the MDA-MB-468 cells with inherent low expression of TLR4 (Fig. 2b). A slight effect of LPS2 was seen in the TLR2/4-negative cell line, CAMA-1, which might represent unspecific binding to other receptors. This was supported by the finding that the mRNA levels of *IL-6* and *IL-8* increased in a similar manner in all TN cell lines except MDA-MB-468, and not in the CAMA cell line (Fig. 3a and data not shown). Interestingly, TLR signaling affected not only the transcription of *IL-6* and *IL-8*, but also the protein translation as judged by cycloheximide (CHX) experiments showing a decreased release of both IL-6 and IL-8 after LPS1 stimulation upon simultaneous treatment with LPS and CHX (Fig. 3b).

### A TLR4-specific DAMP induces pro-inflammatory cytokines in breast cancer cells

We further investigated whether DAMPs could induce TLR2/4-signaling in TN cell lines (MDA-MB-231, SUM-149 and SUM-159) and found that LPS (LPS1 and LPS2), but not the endogenous DAMP HMGB1, significantly induced IL-6 and IL-8 release in MDA-MB-231 cells and SUM-159 cells (Fig. 3c and e), whereas in SUM-149 cells LPS2 induced IL-6 and IL-8 release primarily (Fig. 3d). This finding might reflect that TLR2/4-induced transcription v/s translation might be differentially regulated in breast cancer cells. IL-1β was used as a positive control. Using dual luciferase assays and an NFκB reporter, we confirmed that LPS stimulation of MDA-MB-231 cells induced activation of NFκB but HMGB1 did not (Fig. 3f). We continued with another cancer-related DAMP reported to be a TLR4 ligand, the S100A9 protein [35]. Indeed, stimulating MDA-MB-231, SUM-149 and SUM-159 cells with rS100A9 for 20 h induced a significant increase in both IL-6 and IL-8 release (Fig. 3g). We also tested whether stimulating with HMGB1 for 20 h would induce cytokine release but with negative results (data not shown). Finally, by introducing the MD2/TLR4 complex (pDUO-MD2/TLR4) in otherwise negative MCF-7 cells, we could see a significant expression of both IL-6 and IL-8 as compared to control MCF-7 cells (Fig. 3h). pDUO-MD2/TLR4 is an expression vector that is designed to co-express the *MD2* and *TLR4* genes needed to interact with each other for functional signaling to occur upon ligand binding [42].

### Constitutive expression of IL-6 and IL-8 is inhibited by silencing of TLR4

**Fig. 4:**
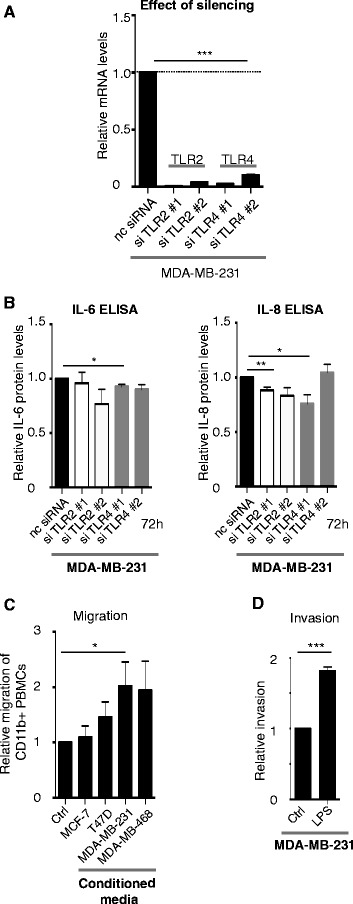
Toll-like receptor 4 (*TLR4*) silencing decreases endogenous levels of pro-inflammatory cytokines. **a** Effect of TLR2/4 silencing in breast cancer cells transfected with negative control (*nc*) siRNA, or siRNA directed against *TLR2* mRNA (*si#1* and *si#2*) or *TLR4* mRNA (si#1 and si#2) was analyzed using quantitative real-time PCR; ***P <0.001 (analysis of variance (ANOVA)). **b** IL-6 (*left*) and IL-8 (*right*) ELISA on supernatants from MDA-MB-231 breast cancer cells transfected with nc siRNA, or siRNA directed against *TLR2* mRNA (si#1 and si#2) or *TLR4* mRNA (si#1 and si#2); n = 4. *Error bars* standard error of the mean (SEM); *P <0.05, **P <0.01, ***P <0.001 (ANOVA). **c** Boyden chamber migration assays. Migration of primary human myeloid cells towards supernatants from different cell lines indicated. Human primary peripheral blood mononuclear cells were isolated as previously described [48] and allowed to migrate through a Costar Transwell® Permeable Support 8.0-μm 24-well plate (Corning) to the supernatants of breast cancer supernatants cultured under serum-free conditions. Percentage of migrated CD11b^+^ cells was analyzed using a flow cytometer and CD11b-APC antibodies (BD Sciences); n = 4. *Error bars* SEM; *P <0.05, **P <0.01, ***P <0.001 (ANOVA). **d** Matrigel invasion assays. Invasion of lipopolysaccharide (LPS)-stimulated/un-stimulated MDA-MB-231 cells into matrigel invasion chambers (BD Sciences) as indicated: 25 × 10^3^ MDA-MB-231 cells were stimulated or not with LPS and allowed to invade from 72 h. Amount of invaded cells was analyzed using crystal violet staining and manual counting in four separate experiments; n = 4. *Error bars* SEM; *P <0.05, **P <0.01, ***P <0.001 (Student’s *t* test)

The impact of TLR4 signaling (possibly by endogenous DAMPs) on the constitutive expression of IL-6 and IL-8 seen in the MDA-MB-231 cells was analyzed. To this end we used negative control (nc) siRNA or siRNA specific for *TLR2* (siTLR2#1 and #2) and *TLR4* (siTLR4#1 and #2) (Fig. 4a), and analyzed the IL-6 and IL-8 levels 72 h post transfection. Both siTLR2 and siTLR4 slightly decreased the endogenous levels of IL-6 and IL-8 (Fig. 4b).

### TLR2/4 expression affects migration and invasion

The TLR2/4-induced pro-inflammatory cytokines can be chemoattractants for myeloid cells. We therefore next investigated whether primary human CD11b^+^ myeloid cells would migrate toward supernatants collected from breast cancer cells with a TN phenotype as compared to ER^+^ breast cancer cells. Indeed, primary human myeloid cells migrated significantly more to supernatants collected from MDA-MB-231 cells as compared to from ER^+^ MCF-7 or T47D cells, but as expected also to the TLR4-negative, but pro-inflammatory cytokine-secreting, MDA-MB-468 cells (Fig. 4c).

Other parameters that might be affected by TLR4 expression in breast cancer cells were also investigated; invasion, apoptosis and proliferation (Fig. 4d and Additional file 2: Figure S1A-B). In summary, invasion into matrigel invasion chambers by MDA-MB-231 breast cancer cells was increased when the TLR2/4 ligand LPS was added to the invading cells (Fig. 4d). Apoptosis in MDA-MB-231 breast cancer cells where TLR2 or TLR4 was silenced gave either conflicting results (TLR2) or was not affected (TLR4) (Additional file 2: Figure S1A), and finally proliferation using ^3^H-incorporation assays of MDA-MB-231 breast cancer cells where TLR4 was silenced was not affected as compared to control (Additional file 2: Figure S1B).

To finally evaluate if other relevant TLRs were functional in the TN breast cancer cells we also performed stimulations of MDA-MB-231 cells with necrotic cell supernatant (NCS; TLR3 ligands [43]). Release of IL-8 but not IL-6 was affected by addition of NCS in a concentration-dependent manner (see Additional file 2: Figure S1C).

### TLR4 is expressed in ER/PR-negative breast cancers and correlates with poor survival

**Fig. 5:**
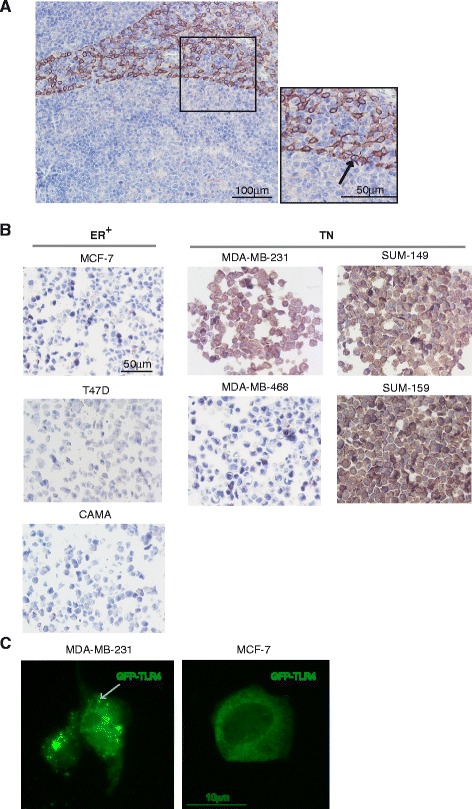
Analysis of Toll-like receptor 4 (*TLR4*) protein expression in tonsil and breast cancer cells lines. Immunohistochemical analysis using an anti-human TLR4-specific antibody on paraffin-embedded tonsil (**a**) or cell pellets from the cell lines indicated (**b**). *Arrow* indicates the membranous staining. **c** MDA-MB-231 cells expressing the TLR co-receptors MD2 and CD14 but not ERα *left*), and MCF-7 cells expressing ERα but not MD2 or CD14 (*right*) were transfected with *GFP*-tagged hTLR4 (pUNOI-hTLR4-GFP; Invivogen) for 48 h. Localization was investigated using immunofluorescence microscopy. The green fluorescent protein (GFP)-tagged hTLR4 was expressed in a vesicular pattern in the cytoplasm of MDA-MB-231 cells and evenly in the cytoplasm of MCF-7 cells. *Arrow* indicates the vesicular pattern. *ER+* estrogen receptor-positive, *TN* triple-negative

TLR proteins are difficult to analyze because the antibody specificity is generally poor. We carefully evaluated several antibodies and found one to be highly specific. This antibody was confirmed first by using human tonsil tissue as positive control, showing the typical pattern of TLR4-expressing cells surrounding the follicles (Fig. 5a). Having optimized IHC, we subsequently stained formalin-fixed and paraffin-embedded cell pellets of the cell lines used in this study. All ER^+^ cell lines were negative for TLR4, whereas three out of four TN cell lines displayed marked cytoplasmic positivity, corroborating our mRNA results (Fig. 5b). The cytoplasmic localization of TLR4 in breast cancer cells was supported by transfection of breast cancer cells using a green fluorescent protein (GFP)-tagged TLR4 plasmid (pUNOI-hTLR4-GFP) (Fig. 5c). We found that in cells expressing both the TLR4 co-receptors MD2 and CD14 (MDA-MB-231 cells; Fig. 5c left) TLR4-GFP was expressed in a vesicular pattern in the cytosol, whereas in breast cancer cells lacking both MD2 and CD14 (MCF-7 cells; Fig. 5c right), TLR4-GFP was expressed evenly in the cytoplasm.

**Fig. 6:**
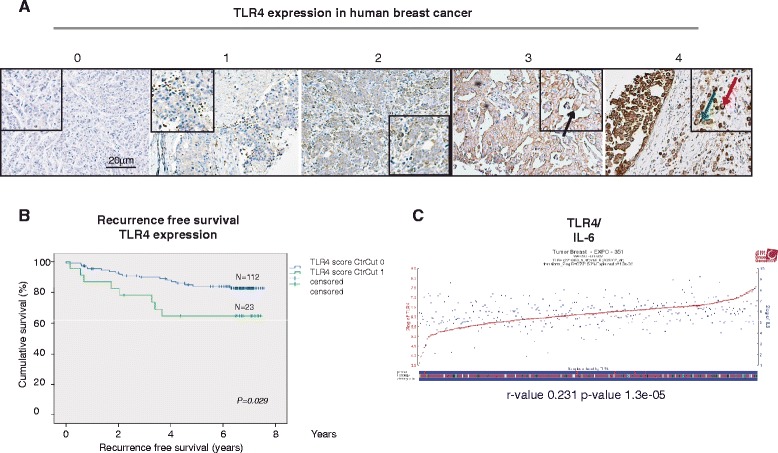
Toll-like receptor 4 (*TLR4*) expression in breast tumors and survival curves. **a** Immunohistochemical TLR4 expression sample images from breast cancer tissue microarray cores, from *left* to *right*: negative (0), weak (1), moderate (2), strong (3) and very strong (4). Boxes with magnification and arrows to indicate localization (*black arrow* membranous, *green arrow* leukocyte, *red arrow* cytoplasmic staining. **b** Kaplan-Meier curves illustrating differences in recurrence-free survival according to TLR4 cytoplasmic expression in breast tumors. Cytosolic scoring parameters were grouped into 0–2 (0) and 3–4 (1) critical cutoffs. **c** Gene expression profile analysis of *TLR4* mRNA in relation to *IL-6* in (R2: microarray analysis and visualization platform [40]; Tumor breast EXPO-351)

A TMA of 144 breast cancer patients was subsequently stained and analyzed for correlation with other histological and clinical parameters. The staining was judged as cytoplasmic staining of intensities 0, 1, 2, 3 and 4 (see “Methods”) (Fig. 6a). When the analysis was performed these cytosolic scoring parameters were grouped into 0–2 (0) and 3–4 (1) critical cutoffs. Table 1 shows the clinical parameters and correlations found with the TLR4-specific antibody. The intensity groups 3–4 (1) correlated significantly with the ER/PR-negative patient group and the basal-like status marker CK5. It did not correlate to Her2 expression (or lack of expression), however (Tables 1, 2 and 3). Tables 2 and 3 specifically show the correlation between Her2 and TLR4. Survival curves using the two cutoff groups indicate that as expected, the group with high TLR4 expression had significantly worse recurrence-free survival (*P* <0.029) (Fig. 6b). Membranous staining was also scored (0, 1) but there was no strong correlation (data not shown).

**Table 1:** Correlation between TLR4 expression and clinicopathologic features in primary breast cancer (n = 144 patients)

	Toll-like receptor 4		
Clinicopathologic features	Correlation coefficient	*P* value (two-tailed)	Number
Age	−0,042	0,629	135
Nodal stage	0,042	0,646	122
Tumor size	−0,006	0,944	135
Ki67	0,003	0,971	117
nhg	0,094	0,276	135
Her2 subtype	0,158	0,072	131
ER status	−0,170	0,049*	135
PR status	−0,206	0,016*	135
CK5	0,184	0,037*	129

*P <0.05 using SPSS and Spearman’s Rho test. *Her2* human epidermal growth factor receptor 2, *ER* estrogen receptor, *PR* progesterone receptor, *nhg* Nottingham histological grade

**Table 2:** Crosstab over Toll-like receptor 4 (TLR4) (0, 1) and Her2 (0, 1)

		TLR4 (0)	TLR4 (1)	Total
Her2	(0)	83	33	116
	(1)	6	6	12

Chi square test value 2.366, *P* = 0.124. *Her2* human epidermal growth factor receptor 2

**Table 3:** Crosstab over Toll-like receptor 4 (TLR4) (0,1) and ER/PR-negative/positive Her2+ tumors

	TLR4 (0)	TLR4 (1)	Total
ER/PR-negative Her2+	1	3	4
ER/PR-positive Her2+	5	3	8

Chi square test value 1.375, *P* = 0.241. *Her2* human epidermal growth factor receptor 2, *ER* estrogen receptor, *PR* progesterone receptor

Finally, using a publicly available data site (R2: microarray analysis and visualization platform [40]; Tumor breast EXPO-351) with gene expression profiles of 351 primary breast cancers, we found positive correlation between expression of *TLR4* mRNA and *IL-6* (*r* value 0.231, *P* = 1.3e-05) (Fig. 6c).

## Discussion

Breast cancers with an ER-negative phenotype have previously been shown to promote a strong pro-inflammatory microenvironment [44]. Furthermore, historically there is a negative relationship between ERα and NFκB that has previously been described in depth [10, 27–30]. Despite the fact that ER signaling can inhibit NFκB activity and vice versa, there is no evidence that the development of ER-negative breast tumors are caused by constitutive NFκB activity. Rather, it may be a result of the typical molecular gene landscapes found in luminal A compared to basal breast cancers, respectively. A link between PRR, e.g., TLR-induced activation of NFκB in breast cancer and its relation to expression of ER, has not been described. Both IL-6 and IL-8 can be highly expressed in TN breast cancers and this has partly been attributed to constitutively active NFκB [44]. In order to investigate whether TLRs, which are known to induce strong activation of NFκB, are expressed primarily in TN breast cancers and if this might affect the expression of pro-inflammatory genes in the same, we investigated the functional role of TLRs and co-receptors in breast cancer.

In immune cells, TLR expression is generally inhibited by prolonged activation of NFκB [45]. In contrast, our findings show that TLRs (TLR2, TLR 3, TLR 4) are preferentially expressed in TN breast cancer cell lines with constitutive NFκB activity, suggesting that the TLRs may be responsible for the NFκB activation pathway rather than induced by the same. Although introduction of a functional MD2/TLR4 complex in an ER^+^ cell line has been shown to induce expression of pro-inflammatory cytokines, silencing of TLR4 in TN cells only caused a slight decrease in pro-inflammatory mediator release, indicating that the constitutive NFκB activation seen in TN cells in general is caused by another mechanism [44]. Apart from MDA-MB-468, the TN breast cancer cells were also demonstrated to express the co-receptors CD14 and MD2 meaning that they harbor the necessary proteins for a functional TLR4 signal to occur [20–22]. The exception, MDA-MB-468, only expressed CD14 and in line with this showed no biological TLR function. In the patient cohort we found correlation between TLR4 expression and ER/PR-negative tumors, but not TN tumors. This strengthens the interrelationship between TLR4, ER and NFκB activity, as expression of HER2 was not correlated in the TLR4-expressing primary tumors. We did not perform our in vitro analyses on any Her2^+^ breast cancer cell line. Interestingly, the typical membrane staining seen in immune cells was not as obvious in the malignant cells, indicating that different regulation of TLR expression and signaling could be possible in cancers. This was previously described in neuroblastoma cells [46] and is also supported by our finding that a GFP-tagged hTLR4 primarily showed a vesicular cytoplasmic localization in breast cancer cells. Furthermore and supporting this observation, it was recently reported that the TLR4-specific DAMP, S100A9, needs to be internalized to be able to signal via TLR4 [15]. Indeed, scoring of membrane TLR4 expression in breast cancer lesions did not reveal as much as that of cytoplasmic staining, and both TLR2 and TLR4 have been reported to be expressed intracellularly as well [47].

The DAMP, HMGB1, has previously been shown to signal via TLR4 in myeloid cells [6, 19]. Although we have also previously shown this in primary myeloid cells [48], we did not see an effect of HMGB1 on breast cancer cells in vitro. This could be due to different culture conditions, or to receptor expression patterns in myeloid as compared to cancer cells which might also reflect the fact that different sources of LPS generate different signals in the different cell lines in this study. Instead, we could show that the DAMP, S100A9, also induced pro-inflammatory proteins in breast cancer cells expressing TLR4.

It has previously been shown that NFκB [29] and targets (IL-6) [49] can downregulate ERα. We also investigated whether overexpression of TLR4 would affect ERα expression per se in ER^+^ MCF-7 cells. We did see a slight although non-significant decrease of ERα after 72 h (data not shown), a finding that is probably explained by the significantly increased levels of IL-6 we observed in these experiments (Fig. 3h). In spite of this, we suggest that the ER/PR-negative breast cancer subtype probably is not caused by expression of TLRs and their downstream mediators, but rather further affected by them. Perhaps the expression of TLRs is even affected by the ERα/FoxA1/GATA3 network [50]. We show that both PAMPs and DAMPs induced release of pro-inflammatory mediators in ER/PR-negative breast cancer cells in vitro, a process that was regulated both at the transcriptional and post-transcriptional level. This means that although ER-negative breast cancer cells express high endogenous levels of pro-inflammatory mediators, a functional TLR4 is still likely to enhance their phenotype and surrounding inflammatory microenvironment, and this is also reflected by the decreased recurrence-free survival seen in the patients with tumors expressing TLR4 at high levels. In support of this, previous studies have shown that TLR4 expression promotes metastasis in a breast cancer model, an effect that was even enhanced by Paclitaxel [25, 26].

## Conclusion

The findings presented in this study suggest that TLR4 is expressed in a functional form in ER/PR-negative breast cancers primarily. We suggest that TLR4 should be viewed as a possible therapeutic target in ER/PR-negative breast cancers to decrease the pro-inflammatory environment and hence the metastatic spread.

## Additional files

Additional file 1: Table S1. Primer sequences. (PDF 94 kb) Additional file 2: Figure S1.
**A** Annexin V staining of MDA-MB-231 cells using flow cytometry to investigate apoptosis of MDA-MB-231 cells transfected with negative control (*nc*) siRNA, or siRNA directed against *TLR2* mRNA (*si#1* and *si#2*) or *TLR4* mRNA (*si#1* and *si#2*). TLR2 (si#1 and #2) gave contradicting results while TLR4 si#1 and #2 gave no effect (n = 3). Error bars indicate standard error of the mean (SEM); **P* <0.05, ***P* <0.01, ****P* <0.001 (analysis of variance (ANOVA)). **B** 3H-incorporation assay using previously published methods [48] to investigate proliferation of MDA-MB-231 cells transfected with negative control (*nc*) siRNA, or siRNA directed against *TLR2* mRNA (*si#1* and *si#2*) or *TLR4* mRNA (*si#1* and *si#2*) (n = 6). Error bars indicate SEM; **P* <0.05 ** *P* < 0.01, ****P* <0.001 (ANOVA). **C** IL-6 (*left*) and IL-8 (*right*) ELISA performed on supernatants from MDA-MB-231 breast cancer cells stimulated with increasing amounts of necrotic cell supernatants (*NCS*): 100 μl = 1:1, 50 μl = 1:4, 25 μl = 1:8 (n = 4). Error bars indicate SEM; **P* <0.05, ***P* <0.01, ****P* <0.001 (ANOVA). (PDF 176 kb)

## Abbreviations

ANOVA: analysis of variance
CBA: cytokine bead array
CHX: cycloheximide
DAMP: damage-associated molecular pattern
ELISA: enzyme-linked immunosorbent assay
FBS: fetal bovine serum
ER: estrogen receptor
GFP: green fluorescent protein
Her2: human epidermal growth factor receptor 2
HMGB1: High mobility group protein B1
IL: interleukin
LPS: lipopolysaccharide
MAPK: mitogen-activated protein kinase
NCS: necrotic cell supernatant
NFκB: nuclear factor kappa B
PAMP: pathogen associated molecular pattern
PR: progesterone receptor
PRR: pattern recognition receptor
RT-qPCR: quantitative real-time PCR
TLR: toll-like receptor
TMA: tissue microarray
TN: triple-negative (ER^−^PR^−^Her2^−^)
TNF: tumor necrosis factor
