# Supplementary material for: Expression of functional toll like receptor 4 in estrogen receptor/progesterone receptor-negative breast cancer
Source: Breast Cancer Res. 2015 Sep 22;17(1):130. doi: 10.1186/s13058-015-0640-x (PMC4578669; doi:10.1186/s13058-015-0640-x)
Supplement: Additional file 1: Table S1. — Primer sequences. (PDF 94 kb) [file 13058_2015_640_MOESM1_ESM.pdf]

Additional file 1: Table S1

Primer sequences

YWHAZ (F: ACTTTTGGTACATTGTGGCTTCAA, R: CCGCCAGGACAAACCAGTAT)
UBC (F: ATTTGGGTCGCGGTTCTTG, R: TGCCTTGACATTCTCGATGGT)
SDHA (F: TGGGAACAAGAGGGCATCTG, R: CCACCACTGCATCAAATTCATG)
TLR2 (F: GCCTCTCCAAGGAAGAATCC, R: TCCTGTTGTTGGACAGGTCA)
TLR3 (F: TGGTTGGGCCACCTAGAAGTA, R: TCTCCATTCCTGGCCTGTG)
TLR4 (F: AAGCCGAAAGGTGATTGTTG, R: CTGAGCAGGGTCTTCTCCAC)
TLR9 (F: CTGCCTTCCTACCCTGTGAG, R: GGATGCGGTTGGAGGACAA)
MD2 (F: CCGATGCAAGTATTTCATACACCTACT, R: CTCCTTGGAATGTAGAAAATGTGC)
CD14 (F: GCCCTTACCAGCCTAGACCT, R: CCCGTCCAGTGTCAGGTTAT)
IL-6 (F: GGCACTGGCAGAAAACAACC, R: GCAAGTCTCCTCATTGAATCC)
IL-8 (F: ACTGAGAGTGATTGAGAGTGGAC, R: AACCCTCTGCACCCAGTTTTC)
