# Supplementary figures and images for: Expression of functional toll like receptor 4 in estrogen receptor/progesterone receptor-negative breast cancer
Source: Breast Cancer Res. 2015 Sep 22;17(1):130. doi: 10.1186/s13058-015-0640-x (PMC4578669; doi:10.1186/s13058-015-0640-x)

Additional file 2: Figure S1

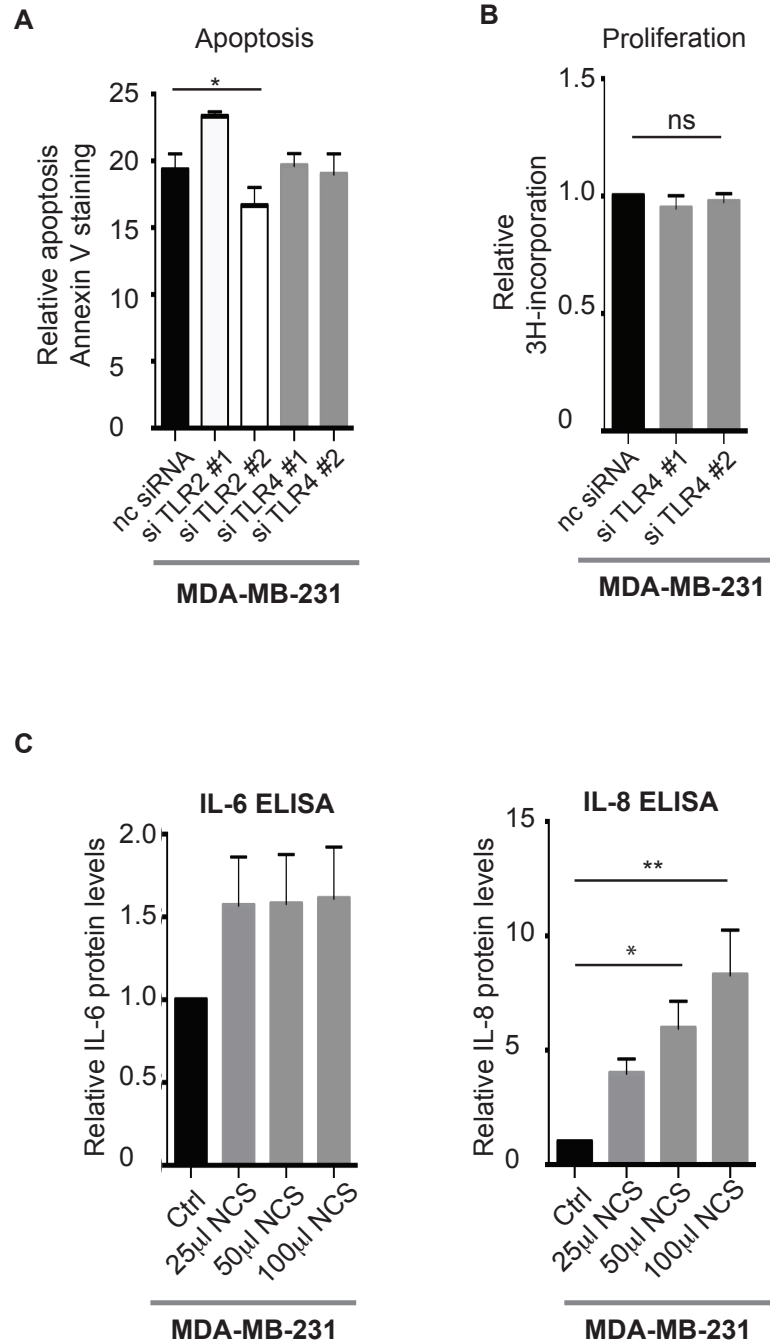

Supplement: Additional file 2: Figure S1. — A Annexin V staining of MDA-MB-231 cells using flow cytometry to investigate apoptosis of MDA-MB-231 cells transfected with negative control (nc) siRNA, or siRNA directed against TLR2 mRNA (si#1 and si#2) or TLR4 mRNA (si#1 and si#2). TLR2 (si#1 and #2) gave contradicting results while TLR4 si#1 and #2 gave no effect (n = 3). Error bars indicate standard error of the mean (SEM); *P <0.05, **P <0.01, ***P <0.001 (analysis of variance (ANOVA)). B 3H-incorporation assay using previously published methods [48] to investigate proliferation of MDA-MB-231 cells transfected with negative control (nc) siRNA, or siRNA directed against TLR2 mRNA (si#1 and si#2) or TLR4 mRNA (si#1 and si#2) (n = 6). Error bars indicate SEM; *P <0.05 ** P < 0.01, ***P <0.001 (ANOVA). C IL-6 (left) and IL-8 (right) ELISA performed on supernatants from MDA-MB-231 breast cancer cells stimulated with increasing amounts of necrotic cell supernatants (NCS): 100 μl = 1:1, 50 μl = 1:4, 25 μl = 1:8 (n = 4). Error bars indicate SEM; *P <0.05, **P <0.01, ***P <0.001 (ANOVA). (PDF 176 kb) [file 13058_2015_640_MOESM2_ESM.pdf]
